# Risk Scoring System based on lncRNA Expression for Predicting Survival in Hepatocellular Carcinoma with Cirrhosis 

**DOI:** 10.31557/APJCP.2020.21.6.1787

**Published:** 2020-06

**Authors:** Jiaxiang Ye, Haixia Li, Jiazhang Wei, Yue Luo, Hongmei Liu, Jinyan Zhang, Xiaoling Luo

**Affiliations:** 1 *Department of Immunology, School of Basic Medical Sciences, Guangxi Medical University, Nanning, China. *; 2 *Department of Otolaryngology and Head and Neck, The People’s Hospital of Guangxi Zhuang Autonomous Region, Nanning, China. *; 3 *Department of Medical Oncology, Guangxi Medical University Cancer Hospital, Nanning, China. *

**Keywords:** Hepatocellular carcinoma, cirrhosis, long non-coding RNA, survival

## Abstract

**Objective:**

This study aims to explore the roles of long non-coding RNAs (lncRNAs) for predicting survival in hepatocellular carcinoma (HCC) patients with cirrhosis.

**Methods:**

A set of lncRNAs differentially expressed between HCC patients with or without cirrhosis was identified using expression profiles of The Cancer Genome Atlas database, and these lncRNAs were screened for their risk scoring system to predict recurrence-free survival (RFS) or overall survival (OS). Predictive ability of risk scoring systems was confirmed using uni- and multivariate Cox analyses while adjusting for clinical features. Predictive lncRNAs were analyzed by functional enrichment analysis.

**Results:**

Our screen identified 22 lncRNAs that were upregulated in the presence of cirrhosis and 59 that were downregulated. To predict OS of HCC patients with cirrhosis, a risk scoring system was developed with four lncRNAs (LINC02086, LINC00880, LINC01549 and AC136475.3); to predict RFS in these patients, the risk scoring system contained five lncRNAs (SH3RF3-AS1, AC104117.3, AC136475.3, LINC00239 and MRPL23-AS1). All risk scoring systems were associated with an area under the receiver operating characteristic curve > 0.7. Based on uni- and multivariate Cox analyses, the risk scoring system could serve as a significant independent predictor for OS in HCC patients with cirrhosis. Functional enrichment analysis suggested that the lncRNAs in the risk scoring systems are involved primarily in the pathway of Wnt signal and cytokine-cytokine receptor interaction.

**Conclusion:**

Risk scoring systems based on lncRNAs can effectively predict OS of HCC patients with cirrhosis. The system should be further developed and validated in larger, preferably multi-site patient populations.

## Introduction

Liver cancer remains the sixth most frequent cancer globally, with approximately 841,000 new diagnosed cases and 782,000 deaths annually. Hepatocellular carcinoma (HCC) is the most frequent common liver cancer, accounting for 75–85% of liver cancers (Bray et al., 2018; Forner et al., 2018; Kulik and El-Serag, 2019). Despite significant improvements in diagnosing and treating HCC, its heterogeneity means that it continues to be associated with relatively low rates of recurrence-free survival (RFS) and overall survival (OS) (Bruix et al., 2014; Fong and Tanabe, 2014). Currently no biomarkers have proven effective for predicting prognosis, reflecting the complicated nature of the disease. Further efforts are needed to identify biomarkers that can predict prognosis and guide treatment. 

Long non-coding RNAs (lncRNAs), located in the cytoplasm and nucleus of eukaryotic cells, are non-coding RNAs longer than 200 nt (Ponting et al., 2009). These molecules play critical roles in the progression and occurrence of malignant cancers (Huarte, 2015; Schmitt and Chang, 2016; Huang et al., 2018; Wu et al., 2018a). For example, lncRNA-TUBB2A and KRTAP5-AS1 compete with endogenous RNAs to modulate the function of claudin-4, affecting prognosis of patients with gastric cancer (Song et al., 2017). The lncRNA lnc-EGFR stimulates T-regulatory cell differentiation, thereby promoting HCC immune evasion (Jiang et al., 2017), while lncRNA-6195 inhibits α-enolase activity and thus HCC progression (Yu et al., 2018). 

An important risk factor for developing HCC is hepatic cirrhosis, which is characterized by the appearance of regenerative nodules surrounded by fibrous bands. Cirrhosis occurs secondary to chronic liver injury, and it can lead to portal hypertension or HCC(Schuppan and Afdhal, 2008). Since the clinical characteristics and prognosis of HCC patients can differ depending on whether cirrhosis is present (Grazi et al., 2003; Gassmann et al., 2010; Wang et al., 2013; Techathuvanan et al., 2015), we wanted to examine whether some lncRNAs are differentially expressed in the presence or absence of cirrhosis, and whether these might influence risk of recurrence or death. Using expression profile data in The Cancer Genome Atlas (TCGA) database, we developed a risk scoring system based on lncRNA levels and showed that it could predict survival of HCC patients with cirrhosis. 

## Materials and Methods

*Datasets on HCC patients*

Expression profiles of lncRNAs and mRNAs from HCC patients, together with the corresponding clinical information, were taken from TCGA (version 09-14-2017 for HCC) via the UCSC Xena server (https://xenabrowser.net/datapages/). Patient data were included in the present study if their HCC was confirmed by histology, complete lncRNA and mRNA expression profiles were available, cirrhosis status was known, and sufficient data were available to determine OS and RFS. After applying these criteria, 77 HCC patients with cirrhosis and 130 without cirrhosis were selected (Table 1). This study was prepared in accordance with TCGA guidelines (https://cancergenome.nih.gov/publications/publication-guidelines). No ethics approval was required for this study since the data came from TCGA.

*Identification of lncRNAs differentially expressed between HCC patients with or without cirrhosis*

After removal of lncRNAs showing zero expression in more than 50% of patients, the “edgeR” package in R (Robinson et al., 2010) was used to identify lncRNAs differentially expressed between patients with or without cirrhosis, defined as those associated with |log2fold change (log2FC)| > 1 and false discovery rate (FDR) < 0.05. Volcano and cluster heat maps were created by “gplots” and “heatmap” packages in R.

*LncRNA–based risk scoring systems*

Firstly, the normalized expression values of multiple samples of the same patient were averaged. Then, univariate Cox analysis was performed to screen differentially expressed lncRNAs for a significant relationship with OS or RFS. Those lncRNAs associated with p < 0.05 were included in subsequent multivariate Cox regression, and the best model was selected using the method of backward stepwise. A risk scoring system was defined using a linear combination of the lncRNA expression levels, each multiplied by a regression coefficient β: 

Risk score = (β1 * lncRNA1 level) + (β2 * lncRNA2 level) + (β3 * lncRNA3 level) + (β4 * …level) + ... 

Using this formula, a risk score was calculated for each patient, and the ability of this score to predict survival was evaluated by time-dependent receiver operating characteristic (ROC) curves in three years. Patients were divided into groups at high or low risk based on the median risk score, and shown on a non-cluster heat map. Kaplan–Meier survival curves were compared between patients at high or low risk. All these analyses were performed using R/Bioconductor (version 3.4.4).

*Validation of the prognostic significance of risk scoring systems*

Uni- or multivariate analyses were used to verify associations between risk score and survival. If these analyses did not return any significant results, stratified analyses were conducted to identify factors that might be affecting the results, based on the chi-squared test. All these analyses were carried out using SPSS 16.0 (IBM, Chicago, IL, USA), and the threshold for significance was a two-sided p < 0.05.

*Co-expression and functional analyses of lncRNA-related mRNAs*

Based on data from the 207 HCC patients, mRNAs whose expression co-varied with that of lncRNAs in the risk scoring system were identified based on a two-sided |Pearson correlation coefficient | > 0.40 and a z-test p < 0.01(Fan and Liu, 2016). These mRNAs were analyzed for Kyoto Encyclopedia of Genes and Genomes (KEGG) pathway enrichment using the “clusterProfiler” package in R (Yu et al., 2012). Differences in enrichment were considered significant when associated with p < 0.05.

## Results

*LncRNAs differentially expressed in the presence or absence of cirrhosis in HCC patients*

**Figure 1:**
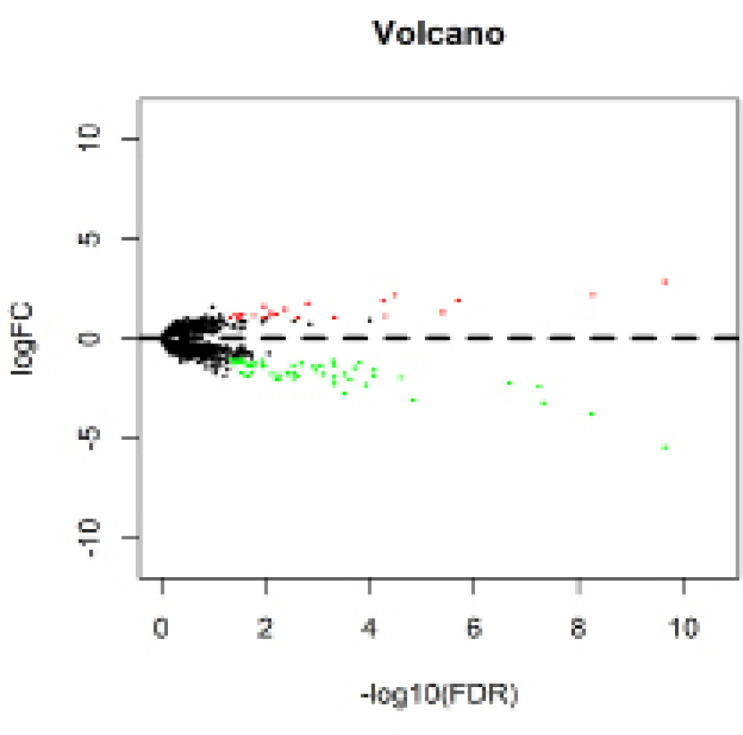
Volcano Map of the Differentially Expressed lncRNAs in HCC Patients between with Cirrhosis or without Cirrhosis. Red spots represent up-regulated genes, and green spots represent down-regulated genes

**Figure 2:**
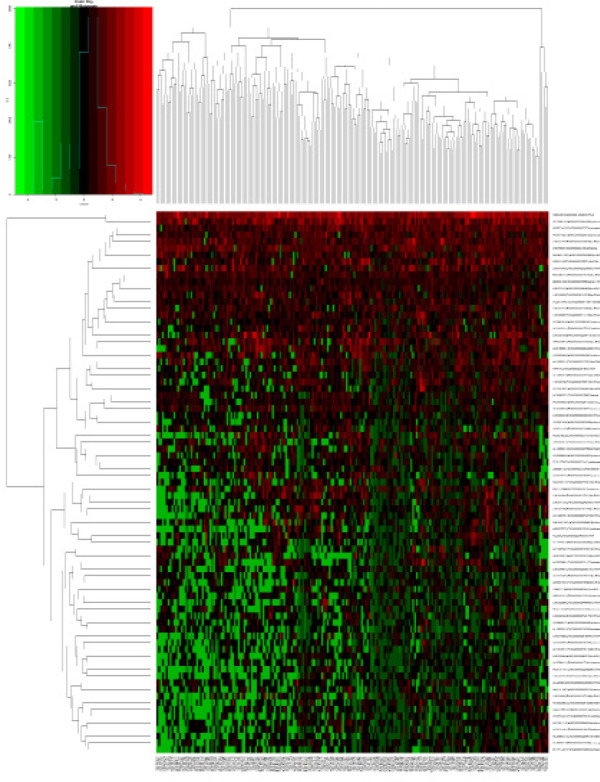
Heatmap Based on the Differentially Expressed lncRNAs in HCC Patients between with Cirrhosis or without Cirrhosis

Our analysis included the lncRNA expression profiles in 77 HCC patients with cirrhosis and 130 HCC without cirrhosis obtained from the TCGA database. A total of 81 differentially expressed lncRNAs were identified, 22 (27.16%) of which were up-regulated in cirrhosis and 59 (72.84%) down-regulated. Table 2 shows the first 20 up- and down-regulated lncRNAs, together with the corresponding values for log2FC, p, and FDR. Figure 1 shows all differentially expressed lncRNAs plotted according to -log10 (FDR) and log2FC, while Figure 2 shows a heatmap indicating the relative specificity of differentially expressed lncRNAs.

*Risk scoring system based on lncRNA expression and OS prediction*

Univariate Cox analysis of patients with cirrhosis identified five lncRNAs (LINC02086, LINC00880, LINC01549, AC136475.3 and HOXA-AS3) significantly associated with OS (p < 0.05). Of these, multivariate regression analyses identified the first four as independent prognostic indicators of OS (Table 3). The resulting risk scoring system was 

Risk score = (0.335 * LINC02086) + (0.372 * LINC00880) + (-0.161 * LINC01549) + (-0.309 * AC136475.3).

In this scoring system, higher expression of LINC02086 and LINC00880 predicted worse OS (β > 0), while higher expression of AC136475.3 and LINC01549 predicted better OS (β < 0). 

**Figure 3:**
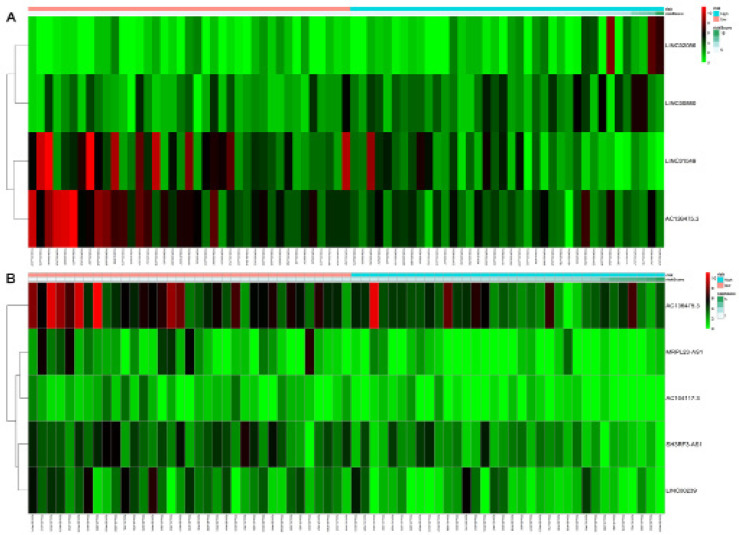
The Non-Cluster Risk Heat Map of Risk Score for OS(A) and RFS (B) in HCC Patients with Cirrhosis. The risk score rises gradually from left to right

**Figure 4.**
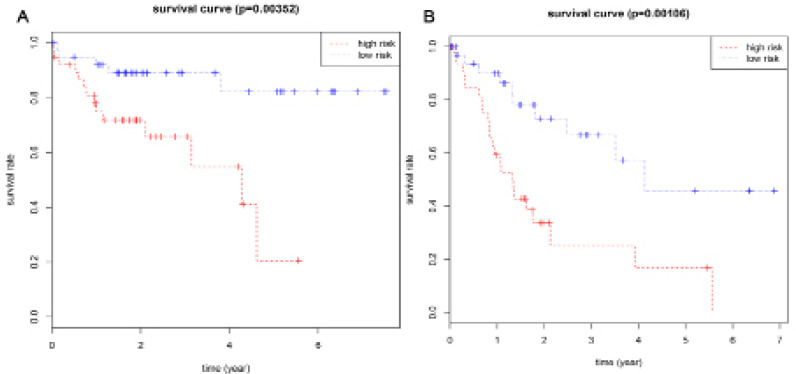
Kaplan-Meier Survival Curves for OS (A) and RFS (B) in HCC Patients with Cirrhosis According to the Risk Cutoff Point

**Figure 5:**
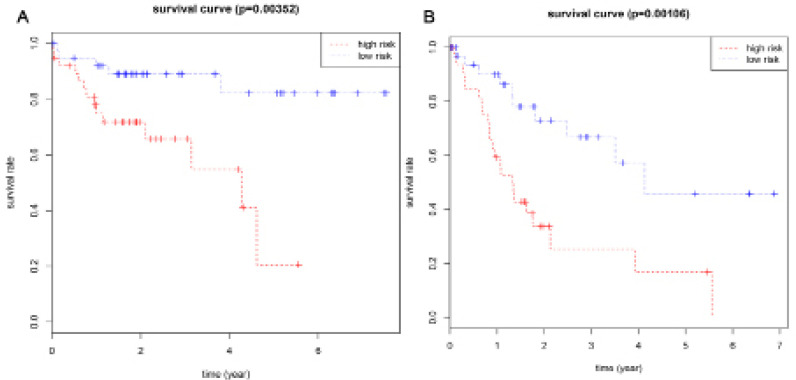
ROC Curves Analysis of the Risk Score for OS(A) and RFS (B) in HCC Patients with Cirrhosis

Based on their risk scores, patients with cirrhosis were classified as being at low or high risk of poor OS using the median risk score as a cut-off (Figure 3A). Kaplan–Meier curves showed that high-risk patients showed significantly lower OS rates at 3 years (65.8% vs 89.1%) and 5 years (20.6% vs 82.2%) (Figure 4A). The area under the ROC curve (AUC) for the risk scoring system was 0.818 (Figure 5A).

After obtaining these promising results, we wished to check whether risk scoring based on lncRNAs could predict OS independently of clinicodemographic characteristics of the patients. This analysis is important given the heterogeneity of clinical presentation of HCC, and the range of factors that can influence prognosis. First we conducted univariate Cox regression to identify which clinical features were significantly associated with OS, and then we subjected this subset of factors to multivariate analysis. 

Univariate analysis of patients with cirrhosis identified risk score and age as significantly associated with OS, but not body mass index (BMI), ethnicity, alpha fetoprotein (AFP), gender, hepatitis, alcohol consumption, cancer status, histology grade, new tumor event, pathology stage, family cancer history, residual tumor, or vascular invasion. Multivariate Cox regression confirmed that age was an independent predictor of poor OS [hazard ratio (HR) 2.86, 95%CI 1.09-7.56], as was risk score (HR 4.08, 95%CI 1.43-11.68) (Table 4).

*Risk scoring system based on lncRNA expression and RFS prediction*

Univariate analysis identified the following 11 lncRNAs as significantly correlated with RFS of patients with cirrhosis: SH3RF3-AS1, AC104117.3, AC136475.3, LINC00239, MRPL23-AS1, LINC00494, LINC01970, MEG3, Z93930.3, MIR9-3HG and TRBV11-2. Multivariate analysis showed the first five to be independent prognostic indicators of RFS (Table 5). The resulting risk scoring system was 

Risk score = (-0.2730 * SH3RF3-AS1) + (-0.2463 * MRPL23-AS1) + (-0.2425 * LINC00239) + (-0.1312 * AC136475.3) + (-0.3609 * AC104117.3), in which higher expression of all five lncRNAs was associated with better RFS (β < 0).

Based on their risk scores, patients with cirrhosis were classified as being at low or high risk of poor RFS using the median risk score as cut-off (Figure 3B). Kaplan–Meier curves showed that high-risk patients showed significantly lower RFS rates at 3 years (25.3% vs 66.8%) and 5 years (16.9% vs 57.3%) (Figure 4B). The AUC of the risk scoring system was 0.819 (Figure 5B).

As we did above for OS, we wished to check whether risk scoring based on lncRNAs could predict RFS independently of clinicodemographic characteristics of the patients. For patients with cirrhosis, our analysis showed that AFP and vascular invasion, but not risk score, correlated significantly with RFS in univariate analysis (Table 6). Frequencies of patients with certain clinicodemographic characteristics were stratified into groups at low or high risk of RFS; this analysis identified significant associations of the risk score with AFP, new tumor event and cancer status (Table 7). 

*Functional analysis of mRNAs related to differentially expressed lncRNAs*

**Figure 6:**
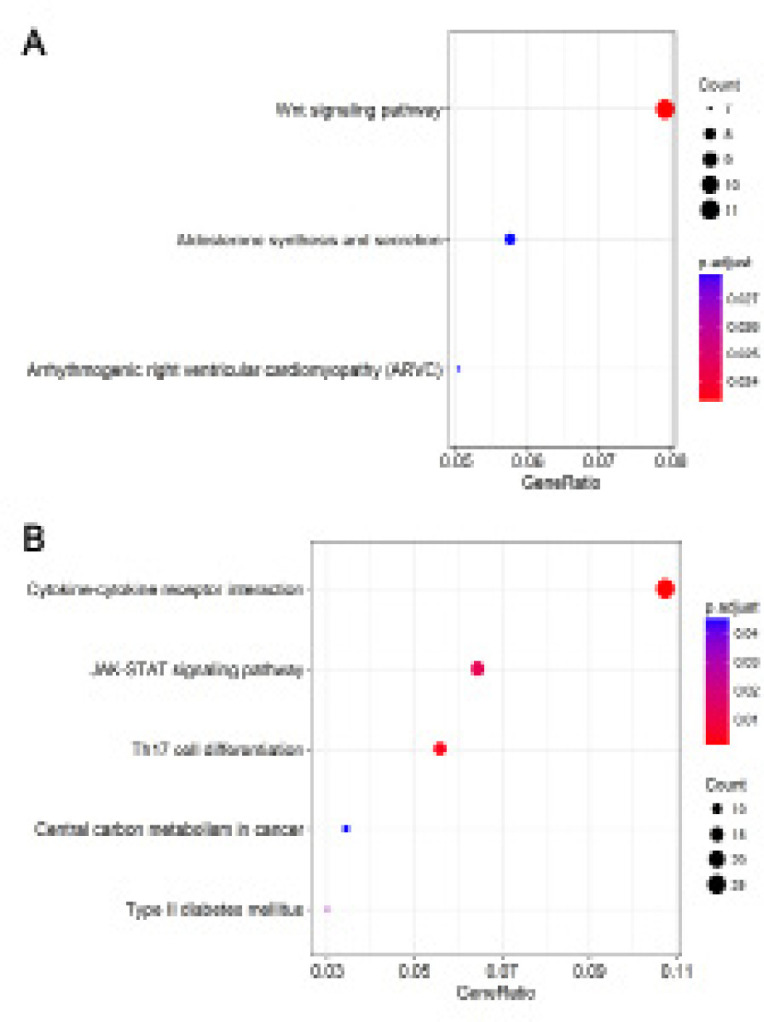
The Top 10 Significantly Enriched Enriched KEGG Pathways of the Highly Related Genes of the lncRNAs of the Risk Scoring Systems for OS(A) and RFS (B) in HCC Patients with Cirrhosis

Correlation in expression levels between the lncRNAs in our risk scoring systems and mRNAs from RNA-seq data was analyzed using Pearson correlation, and several related mRNAs were identified (Supplementary Tables 1-2). These related mRNAs were analyzed using the KEGG signal pathway databases (Figure 6). Functional enrichment analysis showed that mRNAs strongly related to the lncRNAs in our risk scoring systems were involved mainly in the pathway of Wnt signal (Supplementary Figure 1) and cytokine-cytokine receptor interaction (Supplementary Figure 2).

## Discussion

HCC is associated with high morbidity and poor prognosis, and the factors that contribute to poor outcomes are likely to vary substantially in the presence or absence of cirrhosis (Grazi et al., 2003; Gassmann et al., 2010; Wang et al., 2013; Techathuvanan et al., 2015). Therefore we developed an lncRNA-based scoring system to assess a patient’s risk of poor OS or RFS in the presence of cirrhosis. Our results establish the potential of four or five lncRNAs to serve as prognostic biomarkers with respective AUCs of 0.818 and 0.819, suggesting reasonable predictive power. 

After adjusting for other clinical variables, uni- and/or multivariate Cox analyses showed the lncRNA-based scoring system to be significantly associated with OS of HCC patients with cirrhosis, suggesting the lncRNA-based scoring system can serve as a significant independent predictor. However, the system for predicting RFS was not significantly associated with RFS in univariate analysis. Therefore, we stratified patients by low or high risk score and found the following factors to influence risk: AFP, new tumor event and cancer status. Our results suggest that these factors should be taken into account when predicting RFS in HCC patients with cirrhosis. Furthermore, other clinical factors also influenced the survival of HCC patients in our sample. Our study showed that age was an independent factor for the prediction of OS in HCC patients with cirrhosis, which was similar with previous studies (Wang et al., 2013; Techathuvanan et al., 2015; Liu et al., 2018). To gain insight into the prognostic significance of our risk scoring systems, we analyzed the molecular functions of genes highly related to these lncRNAs. We found that the lncRNAs associated with prognosis in HCC are involved mainly in Wnt signaling and cytokine-cytokine receptor interactions.

Several previous studies constructed risk scoring systems to predict the prognosis of HCC patients (Gu et al., 2018; Liao et al., 2018; Ma et al., 2018; Shi et al., 2018; Sui et al., 2018; Wu et al., 2018b; Zhao et al., 2018; Bai et al., 2019; Yan et al., 2019; Ye et al., 2019; Zhang et al., 2019). However, all these risk scoring systems are based on lncRNAs differentially expressed between HCC and normal samples, while our systems are based on lncRNAs differentially expressed between HCC patients with or without cirrhosis. Therefore, our systems may be more specific for HCC involving cirrhosis. One previous study identified a four-lncRNA signature as a prognostic indicator in cirrhotic HCC (Ma and Deng, 2019), with no overlap with our four or five-lncRNA signatures. Those authors extracted their data from a different expression profile dataset, and they used only stratified analyses based on clinical characteristics to validate the prognostic significance of their risk scoring system. In contrast, we applied multivariate Cox regression, which may be more rigorous. This may help explain why none of the lncRNAs in our risk scoring systems occurs in the previously published risk scoring systems for HCC prognosis. The importance of developing prognostic tools for HCC with cirrhosis is underscored by the observation of several microRNAs differentially expressed between patients with or without cirrhosis, particularly mir-149 (Mei et al., 2018), miR-24 and miR-27a (Salvi et al., 2013). These differences may help explain the different prognosis in the two types of HCC. Together, these studies suggest that non-coding RNAs may hold the key to unlocking new strategies in the diagnosis, treatment and management of HCC in the presence of cirrhosis. 

Our results should be interpreted with caution in light of several limitations. First, we could not include type of HCC treatment in our multivariate Cox regression for lack of data, and this variable may affect OS and RFS. Second, some Cox analyses may have been biased because certain clinical data were unavailable for some patients. Third, although the AUC was 0.819 for the system to score risk of poor RFS in HCC patients with cirrhosis, univariate Cox analysis showed that the risk score was not a significant independent predictive factor of HCC prognosis. This model, in particular, should be re-examined carefully with a larger dataset. Last but not least, we could not divide the samples into training and test dataset, which may result in some bias. Indeed, future work should validate all our findings in larger, preferably multi-site patient populations. 

In conclusion, we have defined lncRNA-based risk scoring systems that can effectively predict OS in HCC patients with cirrhosis. Our work highlights the potential usefulness of lncRNAs as prognostic biomarkers, and it provides several leads for the development of novel therapies.

**Table 1:** Clinicopathological Characteristics of 207 HCC Patients with or without Cirrhosis

Clinicopathological characteristics		Patients(n=207)	
		n	%
Age	≤60	96	46.4
	>60	111	53.6
BMI	<25	94	45.4
	≥25	103	49.8
	Not reported	10	4.8
Race	Non-Asian	121	58.5
	Asian	80	38.6
	Not reported	6	2.9
AFP	≤20ng/mL	109	52.7
	>20ng/ml	76	36.7
	Not reported	22	10.6
Gender	Female	66	31.9
	Male	141	68.1
Hepatitis B or C	NO	94	45.4
	YES	104	50.3
	Not reported	9	4.3
Alcohol consumption	NO	148	71.5
	YES	50	24.2
	Not reported	9	4.3
Cirrhosis status	NO	130	62.8
	YES	77	37.2
Histologic grade	G1-2	133	64.2
	G3-4	72	34.8
	Not reported	2	1.0
New tumor event	NO	97	46.9
	YES	101	48.8
	Not reported	9	4.3
Pathologic stage*	Stage I+II	155	74.9
	Stage III+IV	41	19.8
	Not reported	11	5.3
Cancer status	Tumor free	116	56
	With tumor	84	40.6
	Not reported	7	3.4
Family cancer history	NO	111	53.6
	YES	68	32.9
	Not reported	28	13.5
Residual tumor	R0	192	92.7
	non-R0	13	6.3
	Not reported	2	1.0
Vascular invasion	Negative	138	66.7
	Positive	60	29
	Not reported	9	4.3

BMI, Body mass Index; AFP, Alpha fetoprotein; *TNM staging systems.

**Table 2:** Differentially Expressed lncRNAs in HCC Ppatients between with Cirrhosis and without Cirrhosis

	Top 20 up-regulated lncRNAs				Top 20 down-regulated lncRNAs		
	logFC	*P*-Value	FDR		logFC	*P*-Value	FDR
MEG3	1.911879	2.84E-09	2.02E-06	AP000439.3	-5.46056	7.87E-14	2.24E-10
LINC01549	1.197689	0.000727	0.037371	AC139749.1	-3.78362	4.00E-12	5.70E-09
C5orf66	1.025117	1.57E-05	0.002357	AC002398.2	-3.23053	4.09E-11	4.67E-08
AC091182.1	1.341751	6.41E-09	4.06E-06	AC023906.5	-2.39771	6.05E-11	5.75E-08
MEG9	2.83443	4.49E-14	2.24E-10	LINC01474	-2.24764	2.67E-10	2.18E-07
BX649601.1	1.124886	1.16E-07	5.09E-05	AC139769.2	-3.10429	2.62E-08	1.49E-05
LINC01214	1.051004	2.18E-06	0.000485	AC104031.1	-1.95623	5.05E-08	2.62E-05
HOXA11-AS	1.473062	3.43E-05	0.004256	AC069431.1	-1.89819	2.27E-07	8.40E-05
AC098869.2	2.185602	2.85E-12	5.43E-09	TH2LCRR	-1.58174	2.36E-07	8.40E-05
LINC01133	1.321311	7.40E-05	0.007962	AL359853.1	-2.33083	3.69E-07	0.000117
LINC01882	1.620611	0.00011	0.010802	Z93930.3	-1.21647	5.25E-07	0.000158
AL117190.1	2.177359	7.04E-08	3.35E-05	SH3RF3-AS1	-1.45216	6.98E-07	0.000199
LINC02086	1.774533	9.62E-06	0.001567	AC148476.1	-2.04117	9.03E-07	0.000245
Z99289.1	1.010418	0.000601	0.032977	AC007099.1	-2.74984	1.22E-06	0.000304
HOXA-AS3	1.220775	0.000419	0.028584	AP006285.1	-1.77935	1.23E-06	0.000304
TRBV11-2	1.045662	0.001038	0.047752	LINC02334	-2.29279	2.05E-06	0.000485
LINC00487	1.137215	0.000192	0.016844	MIR9-3HG	-1.90608	2.24E-06	0.000485
AC122108.2	1.167812	7.02E-05	0.007735	LINC01970	-1.30815	2.29E-06	0.000485
AC073115.1	1.884735	1.37E-07	5.57E-05	AL445493.2	-1.97111	2.40E-06	0.000489
AC104051.2	1.141559	0.000478	0.029645	ADIRF-AS1	-1.12681	2.62E-06	0.000516

logFC, log2fold change; FDR, false discovery rate

**Table 3:** Four lncRNAs Correlated with OS of HCC Patients with Cirrhosis in the Best Statistical Model

lncRNA	β	HR	z	*P*-value
LINC02086	0.335	1.398	2.51	0.012
LINC00880	0.372	1.451	2.42	0.015
LINC01549	-0.161	0.851	-1.39	0.165
AC136475.3	-0.309	0.734	-1.82	0.068

HR, Hazard ratio

**Table 4:** Univariate and Multivariate Cox Regression Analysis for OS in HCC Patients with Cirrhosis

	Univariate Cox regression				Multivariate Cox regression			
Variables	*P*-value	HR	95% CI		*P*-value	HR	95% CI	
Risk score (high/low)	0.02	7.11	1.42	35.54	0.01	4.08	1.43	11.68
Age (>60/≤60)	0.02	7.34	1.3	41.57	0.03	2.86	1.09	7.56
BMI	0.21							
<25		Reference						
≥25		0.48	0.07	3.13				
Not reported		25.56	0.37	1750.1				
Race	0.26							
Non-Asian		Reference						
Asian		0.25	0.05	1.31				
AFP	0.34							
≤20ng/mL		Reference						
>20ng/ml		2.8	0.66	11.81				
Not reported		0.44	0.01	15.3				
Gender(Male/Female)	0.44	2.15	0.3	15.11				
Hepatitis B or C	0.1							
No		Reference						
Yes		0.17	0.03	0.87				
Not reported		0.28	0.01	10.28				
Alcohol consumption								
No	0.12	Reference						
Yes		0.12	0.01	1.74				
Histologic grade	0.06							
G1-2		Reference						
G3-4		9.6	1.49	61.93				
New tumor event	0.79	0.69	0.05	10.32				
Pathologic stage*	0.7							
Stage I+II		Reference						
Stage III+IV		2.3	0.33	16.02				
Cancer status	0.37							
Tumor free		Reference						
With tumor		2.11	0.12	35.83				
Not reported		0.11	0	8.67				
Family cancer history	0.25							
No		Reference						
Yes		3.11	0.46	20.93				
Not reported		8.42	0.48	147.11				
Residual tumor	0.7							
R0		0.66	0.08	5.71				
Vascular invasion	0.21							
Negative		Reference						
Positive		3.61	0.87	14.91				

BMI, Body mass index; AFP, Alpha fetoprotein; HR, Hazard ratio; CI, Confidence interval; *TNM staging systems.

**Table 5:** Five lncRNAs Correlated with RFS of HCC Patients with Cirrhosis in the Best Statistical Model

lncRNA	β	HR	z	*P*-value
SH3RF3-AS1	-0.273	0.7611	-2.12	0.034
MRPL23-AS1	-0.2463	0.7817	-1.76	0.078
LINC00239	-0.2425	0.7847	-1.99	0.047
AC136475.3	-0.1312	0.877	-1.46	0.143
AC104117.3	-0.3609	0.6971	-1.73	0.083

HR, Hazard ratio

**Table 6:** Univariate Cox Regression Analysis for RFS in HCC Patients with Cirrhosis

Variables	*P*-value	HR	95% CI	
Risk score (high/low)	0.92	0.93	0.23	3.79
Age(>60/≤60)	0.11	0.33	0.08	1.3
BMI	0.94			
<25		Reference		
≥25		0.72	0.1	4.95
Not reported		390.28	0	2.21E+173
Race	0.35			
Non-Asian		Reference		
Asian		0.22	0.03	1.68
Not reported		23.5	0	3.52E+226
AFP	0.03			
≤20ng/mL		Reference		
>20ng/ml		4.48	0.45	44.66
Not reported		45.46	2.18	947.68
Gender (Male/Female)	0.52	2.6	0.14	47.19
Hepatitis B or C	0.3			
No		Reference		
Yes		0.35	0.07	1.61
Not reported		0.13	0.01	2.63
Alcohol consumption (Yes/No)	0.41	0.42	0.05	3.27
Histologic grade	0.32			
G1-2		Reference		
G3-4		5.29	0.61	45.68
Not reported		67.46	0	1.27E+173
New tumor event	0.66			
No		152118.71	0	3.36E+28
Yes		Reference		
Pathologic stage*	0.1			
Stage I+II		Reference		
Stage III+IV		5.34	1.16	24.58
Not reported		0.27	0	4.97E+170
Cancer status	0.1			
Tumor free		Reference		
With tumor		6.89	1.17	40.49
Not reported		1.46	0	.
Family cancer history	0.23			
No		Reference		
Yes		1.21	0.09	15.49
Not reported		0.11	0.01	1.38
Residual tumor	0.71	0.58	0.04	9.56
Vascular invasion	<0.05			
Negative		Reference		
Positive		3.4	0.59	19.61
Not reported		0.07	0	6.845

Note: BMI, Body mass index; AFP, Alpha fetoprotein; HR, Hazard ratio; CI, Confidence interval; *TNM staging systems.

**Table 7:** Stratified Analyses for the Risk Score of RFS in HCC Patients with Cirrhosis Using Chi-Square Test

	Risk score		*P*-value
Variables	low-risk (n)	high-risk (n)	
Age			0.19
≤60	23	17	
>60	12	17	
BMI			0.9
<25	17	17	
≥25	16	17	
Race			0.81
Non-Asian	15	16	
Asian	19	18	
AFP			0
≤20ng/mL	16	26	
>20ng/ml	18	5	
Gender			0.28
Female	10	6	
Male	25	28	
Hepatitis B or C			0.82
No	9	9	
Yes	26	23	
Alcohol consumption			0.85
No	28	25	
Yes	7	7	
Histologic grade			0.39
G1-2	22	24	
G3-4	13	9	
New tumor event			0
No	25	11	
Yes	10	23	
Pathologic stage*			1
Stage I+II	29	29	
Stage III+IV	4	4	
Cancer status			0
Tumor free	27	13	
With tumor	7	21	
Family cancer history			0.49
No	20	21	
Yes	10	7	
Residual tumor			0.64
R0	33	30	
non-R0	2	4	
Vascular invasion			0.08
Negative	27	19	
Positive	7	13	

BMI, Body mass index; AFP, Alpha fetoprotein; *TNM staging systems

## Appendix

Supplementary Table 1. LncRNA-mRNA co-expression analysis for OS in HCC with cirrhosis.

Supplementary Table 2. LncRNA-mRNA co-expression analysis for RFS in HCC with cirrhosis.

Supplementary Figure 1. Wnt signaling pathway

Supplementary Figure 2. The pathway of cytokine-cytokine receptor interaction
